# Immune Checkpoint FGL1 Expression of Circulating Tumor Cells Is Associated With Poor Survival in Curatively Resected Hepatocellular Carcinoma

**DOI:** 10.3389/fonc.2022.810269

**Published:** 2022-02-22

**Authors:** Qing Yan, Hao-Ming Lin, Ke Zhu, Yi Cao, Xiao-Lin Xu, Zi-Yu Zhou, Lei-bo Xu, Chao Liu, Rui Zhang

**Affiliations:** ^1^Guangdong Provincial Key Laboratory of Malignant Tumor Epigenetics and Gene Regulation and Department of Biliary-Pancreatic Surgery, Sun Yat-sen Memorial Hospital, Sun Yat-sen University, Guangzhou, China; ^2^Department of Hepatic Surgery, The First People’s Hospital of Foshan, Foshan, China; ^3^Department of Emergency, Sun Yat-sen Memorial Hospital, Sun Yat-sen University, Guangzhou, China; ^4^Department of Ultrasound, Sun Yat-sen Memorial Hospital, Sun Yat-sen University, Guangzhou, China

**Keywords:** circulating tumor cells, FGL1, hepatocellular carcinoma (HCC), immunotherapy, clinical application

## Abstract

LAG-3 is one of the common tumor immune checkpoints. LAG-3 can inhibit the activation and proliferation of T cells, and can also suppress immunity by regulating other immune-related cell functions. FGL1 was recently discovered to be the main ligand of immune checkpoint LAG-3 and play a critical role in the inhibition of T cells. However, the FGL1 expression in circulating tumor cells (CTCs) and its clinical significance in hepatocellular carcinoma (HCC) remain unclear. Therefore, this bioinformatics analysis was performed to assess the expression of FGL1 in various tumors and its association with immune infiltration. After that, CTCs from 109 HCC patients were detected and the immunofluorescence staining was performed (CD45, EpCAM, CK8/18/19, Vimentin, Twist, DAPI and FGL1). Then, we investigated FGL1 expression and EMT of CTCs and analyzed its relationship with patient survival and clinical relevance. Bioinformatic results showed that FGL1 expression was abnormal in various tumor and it was correlated with the infiltration level of several immune cells. FGL1 expression was detected in CTCs of 40 patients (36.7%). The proportion of advanced TNM stage (P<0.001) and distant metastasis(P=0.020) in FGL1 positive patients was higher than that of FGL1 negative patients. In addition, patients with FGL1 positive circulating tumor cells had worse postoperative survival than FGL1 negative patients (p=0.0297). The mixed phenotypic CTC presented a higher level of FGL1 expression than any other types, the number of which also predicted worse prognosis(p=0.0443). We also found that the expression of FGL1 on CTCs was associated with the level of FGL1 in tumor tissues. Of 12 patients receiving PD-1/PD-L1 blockade in a total of 109 cases, 8 out of 10 patients with FGL1 positive CTC showed immunotherapy resistance. It is the first study that suggested FGL1 expression in CTCs as an indicator of the poor prognosis in HCC patients. CTC detection may serve as a promising replacement for determination of tumor tissue FGL1 expression and provide evidence for the application of immunotherapy.

## Introduction

Hepatocellular Carcinoma (HCC) is currently ranked the sixth in the world in incidence and the third in mortality among tumors (1, 2). The poor overall prognosis of HCC patients is related to advanced stage at diagnosis and the tendency of recurrence. It is reported in the literature that the 5-year recurrence rate after surgery is still as high as 70% (3). The survival of HCC patients is extremely poor, and new treatments are urgently needed to improve the prognosis of patients.

In recent years, research on immune checkpoints inhibitors (ICIs) of HCC has made many improvements (4, 5). Studies have shown that blocking the PD-1/PD-L1 pathway can significantly improve the prognosis of patients with advanced HCC (6–8). Thus, the bright prospects and great significance of immunotherapy for HCC can never be overemphasized.

However, there are still some shortcomings in immunotherapy for HCC. The most notorious one is the low response rate, which is lower than 20% in HCC patients receiving the PD-1/PD-L1 blockage (4). The second is the immunotherapy resistance of PD-1/PD-L1 inhibitors, which mainly includes primary and acquired resistance (9, 10). Studies have indicated that the combination of ICIs with other drugs and the combination of multiple ICIs could significantly improve the efficacy of HCC immunotherapy (11–13). The future of immunotherapy for HCC lies in the discoveries of new immune checkpoints and the development of treatment strategy combining ICIs with other methods.

In 2019, Wang et al. reported for the first time that FGL1, as the main ligand of Lymphocyte-activation gene 3 (LAG-3), can inhibit the function of T cells by combining with it (14). In addition, the study also found that lung cancer patients with high levels of FGL1 in plasma have relatively poor therapeutic effects of PD-1 inhibitors, suggesting an underlying association between the FGL1/LAG-3 and PD-1/PD-L1 immunotherapy resistance. Gong et al. demonstrated that in breast cancer, the use of nanoparticle-encapsulated si-FGL1 can enhance the anti-tumor immune effect mediated by T cells, and the use of nanoparticles to simultaneously block FGL1 and PD-L1 is effective in enhancing T cell anti-tumor ability and has obvious synergistic effects (15). FGL1 is of great significance as a new immune checkpoint target. Targeting FGL1 therapy or combined with PD-1/PD-L1 antibodies can enhance the effect of anti-tumor therapy.

Studies have shown that PD-1/PD-L1 inhibitors can achieve better results in patients with high PD-L1 expression in tumor tissues. In clinical applications, pathological sections and liver puncture tissue are often used to detect the proportion of HCC cells expressing PD-L1. However, obtaining tissue samples could be difficult in clinical practice. Surgical biopsy is not suitable for patients who cannot undergo surgery. Furthermore, it can’t be neglected that there is a certain risk of bleeding and metastasis through liver biopsy. Moreover, due to the heterogeneity within the tumor, the results of the puncture may not reflect the expression of immunotherapy markers in the whole tumor, which is regarded as false-negative. Therefore, it is necessary to find new and simpler methods to detect the expression of patient immunotherapy markers.

Circulating Tumor Cells (CTCs) are tumor cells derived from the primary tumor found in the blood (16). Therefore, compared with some existing inspection methods, CTC can provided more information about genes, RNA, and proteins changes that represents the specificity of the primary tumor, in line with the current concept of individualized and precise treatment of tumor (17–19). Janning et al. reported that in non-small cell lung cancer, the detection of the PD-L1 level of circulating tumor cells can predict the patient’s response to PD-L1 inhibitors, and the increased expression of PD-L1 levels of circulating tumor cells in patients after treatment can reflect the patient’s response The resistance of PD-L1 (20).

This article detected the FGL1 expression of different types of circulating tumor cells in patients with HCC, analyzed the relationship between the FGL1 expression of circulating tumor cells and the patient’s clinicopathological characteristics and prognosis, and investigates whether circulating tumor cells can be used as a substitute for tissue samples to assist in the clinical selection of patients who may benefit from immunotherapy for HCC.

## Materials and Methods

### Bioinformatic Analysis

The data of FGL1 expression in various types of tumors were from The Cancer Genome Atlas (TCGA) database (https://portal.gdc.cancer.gov/). The differential expression analysis was performed in the TIMER 2.0 platform (http://timer.comp-genomics.org/) (21).

### Clinical Samples

5 ml peripheral blood of 109 HCC patients admitted to our hospital from January 2017 to December 2020 was collected to detect the circulating tumor cells and its FGL1 gene expression. At the same time, the relevant clinical data of these 109 patients with HCC were collected, and all the patients had signed the informed consent form.

The inclusion criteria of this study were:(1)18-80 years old; (2) Circulating tumor cell detection was performed after admission; (3) The clinical data of the patients were complete and available; (4) No radiotherapy or chemotherapy were performed before the operation. The exclusion criteria of this study were:(1) It was combined with other malignant tumors; (2) The amount of peripheral blood collected is insufficient or the quality is inferior;

Patients were followed up regularly after surgery, and overall survival was defined as the date of surgery to the date of the patient’s death or the last follow-up. Follow-up was carried out 1 month after surgery, and every 3 months thereafter. The follow-up period ended on December 31, 2020.

### Canpatrol^®^ Circulating Tumor Cell Detection Technique

5 ml of peripheral blood was collected and the red blood cells were removed. Then, an 8μm pore size filter membrane was used to trap circulating tumor cells and some white blood cells on the filter membrane. After that, the specific probe working solution and amplification working solution were used to incubate the membrane (see **Table S1** for probe sequence). Then we utilized a fluorescently labeled probe to hybridize with the amplified probe to generate a fluorescent signal. The system automatically recognized and captured the fluorescent signal, and interpreted the test results. The method for determining CTC classification was shown in **Table 1** (22). And according to the strength of FGL1 immunofluorescence, the expression level of FGL1 gene is calculated, and it is divided into no expression, low expression, medium expression and high expression.

**Table 1:** Circulating tumor cell classification method.

Cell type	CD45 probe	Epithelial Marker		Mesenchymal Marker	
		EpCAM	CK8/18/19	Vimentin	Twist
Epithelial CTC	–	+	+	–	–
Mixed CTC	–	+	+	+	+
Mesenchymal CTC	–	–	–	+	+
White blood cell	+	–	–	–	–

### Immunohistochemistry

Paraffin slices were baked in 55°C oven for 1h and then deparaffinized and rehydrated. 0.3% H2O2 was used to eliminate endogenous peroxidase activity; Then, slices were placed in EDTA repair solution (PH9.0), under high pressure repair for 15 minutes; in the next step, the primary antibody was added for incubation overnight in a refrigerator at 4°C; The next day, rinsed with PBS for 5min×4 times and added the secondary antibody HRP dropwise, incubated at 37°C for 30min; Added the color developer DAB, removed the section until the tissue was obviously brown stained. then hematoxylin counterstaining for 2min; Paraffin oil was used to seal the sheet.

### Statistical Analysis

Use SPSS statistical software (IBM Statistics 25.0) and GraphPad Prism 8.0 software to analyze and process the obtained data. The Pearson Chi-Square method and Fisher’s Exact Test were used for statistical analysis of categorical variables, and the results were expressed as the number of cases and percentages. Kaplan-Meier method was used for survival analysis, and log-rank test was used to compare survival curves. Paired T test and non-parametric test were used to compare continuous variables between the two groups. P<0.05 was used as the judgment index for statistical difference. ImageJ software were used to analyze the immunohistochemistry results.

In addition, this study also uses the method of propensity score matching (23) to match the relevant clinical indicators of patients between the two groups to reduce the impact of potential confounding factors on prognostic survival analysis.

## Results

### The Result of Bioinformatic Analysis

**Figure 1:**
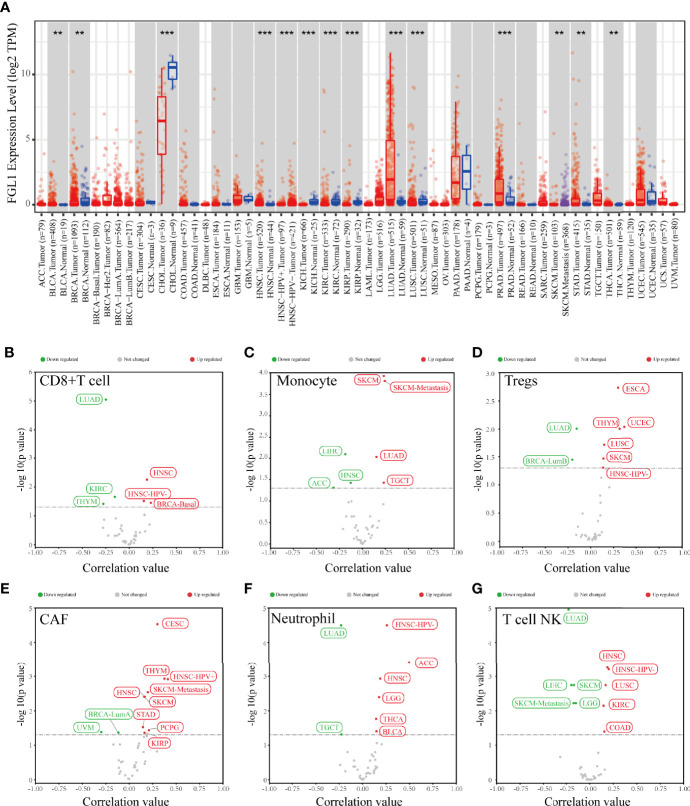
The result of bioinformatic analysis. **(A)** The expression level of FGL1 in various tumors and paired normal tissues. **(B–G)** FGL1 was correlated with infiltrating levels of different immune cells in various tumor tissues. **p < 0.01; ***p < 0.001.

The differential expression analysis result showed that FGL1 expression level in tumor tissue compared to normal tissue was various among several tumors (**Figure 1A**). Also, FGL1 was correlated with the infiltration level of CD8+T cells, monocytes, regulatory T cells (Tregs), carcinoma-associated fibroblasts (CAFs), Neutrophils and Natural killer T cell in various tumors (**Figures 1B–G**). Notably, FGL1 was negatively associated with the infiltration of monocytes and NKT cells in liver hepatocellular carcinoma (**Figures 1C, G**).

### The Result of Circulating Tumor Cells Detection

**Figure 2:**
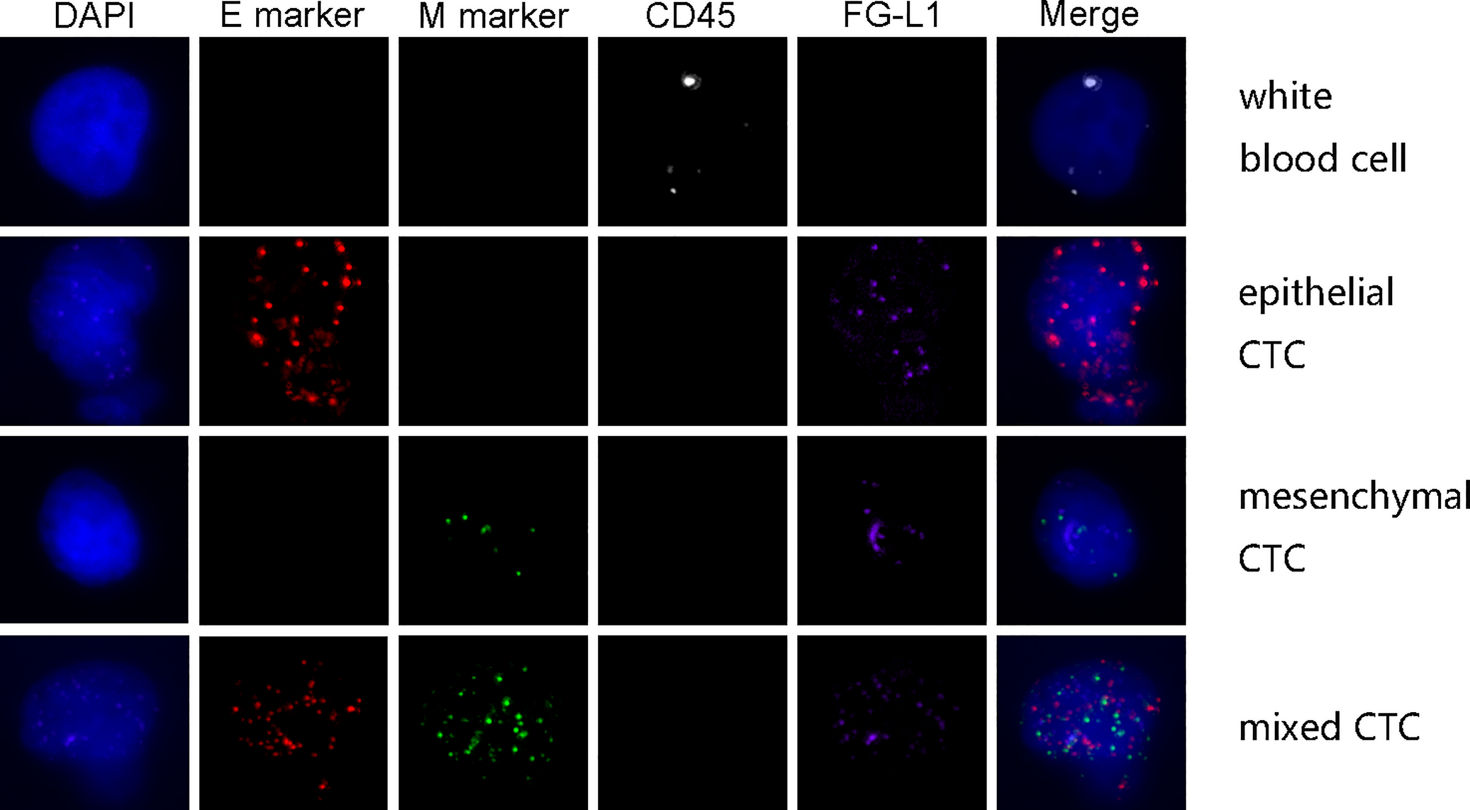
The example of multiple immunofluorescence staining of circulating tumor cells.

A total of 109 patients with HCC were included in this study. Their clinical characteristics were shown in **Table 2**. Among these 109 patients, CTCs were detected in 100 patients(91.7%). The mean value of CTC was 9.94 per patients, and the median value was 6 per patient. Out of 1084 CTC cells from these 109 patients with HCC, 238 (22.0%) were epithelial circulating tumor cells, while 688 (63.5%) mixed circulating tumor cells, and 158 (14.5%) interstitial circulating tumor cells. An example of multiple immunofluorescence results for circulating tumor cell detection was shown in **Figure 2**.

**Table 2:** Clinical characteristics of patients.

Characteristics	Patients (n=109)	
	n	%
Age		
<50	40	36.7
≥50	69	63.3
Gender		
Male	96	88.1
Female	13	11.9
HbsAg		
positive	87	79.8
negative	22	20.2
AFP(ng/ml)		
<400	75	68.8
≥400	34	31.2
Tumor diameter		
<5cm	52	47.7
≥5cm	57	52.3
Tumor lesion		
Single	82	75.2
Multiple	27	24.8
Lymph node metastasis		
Yes	9	8.3
No	100	91.7
Metastasis		
Yes	8	7.3
No	101	92.7
TNM stage		
I-II	74	67.9
III-IV	35	32.1

FGL1 expression was detected in circulating tumor cells of 40 patients (36.7%). The types of circulating tumor cells and FGL1 expression detected in these 40 patients were shown in **Table S3**, including 96 (18.6%) epithelial cells, 333 (64.4%) mixed cells, 88 (17%) mesenchymal cells. Among patients in early TNM stage, 18 patients (24.32%) were FGL1 positive, 56 (75.68%) were FGL1 negative; In patients with advanced-stage tumor, 22 (62.86%) were FGL1 positive patients (62.86%), and 13 were FGL1 negative (37.14%).

### Patients With FGL1 Positive Circulating Tumor Cells Have a Worse Prognosis

Patients were divided into two groups according to whether circulating tumor cells express FGL1, 40 and 69 cases for positive and negative group, respectively. The differences in clinical indicators between the two groups were shown in **Table 3**. The results of data analysis showed that the proportion of patients in the FGL1 positive group with advanced TNM stages (stage III-IV) was higher than that in negative group (P<0.001). In addition, patients in the FGL1 positive group had a higher proportion of distant metastases than the negative group (P=0.020). There was no statistical difference in other clinical characteristics between the two groups.

**Table 3:** Comparison of clinical characteristics.

Indicators	FGL1 Negative	FGL1 Positive	P value
	n=69	n=40	
Age			
≥50Y	47	22	0.171
<50Y	22	18	
Gender			
Male	62	34	0.451
Female	7	6	
HbsAg			
Positive	56	31	0.646
Negative	13	9	
AFP			
≥400 ng/ml	18	16	0.131
<400 ng/ml	51	24	
Tumor diameter			
<5cm	35	22	0.667
≥5cm	34	18	
Tumor lesion			
Single	53	29	0.615
Multiple	16	11	
Lymph node Metastasis			
Yes	4	5	0.284
No	65	35	
Metastasis			
Yes	2	6	**0.020**
No	67	34	
TNM stage			
I-II	56	18	**<0.001**
III-IV	13	22	

Bold values means statistical significance (p < 0.05).

The previous analysis showed that patients with FGL1 positive had a higher proportion of distant metastases and advanced TNM stages. The result of univariate and multivariate COX regression was shown in supplementary tables. In order to determine whether FGL1 positive circulating tumor cells is an independent risk factor affecting the patient’s prognosis, we used the propensity score matching method to avoid confounding factors including age, gender, hepatitis B virus infection, AFP level, tumor number, tumor diameter, lymph node metastasis, TNM stage and distant metastasis. A total of 26 groups of patients were obtained after 1:1 matching of CTC FGL1 positive and negative patients. There was no significant difference in clinical indicators between the two groups after matching.

**Figure 3:**
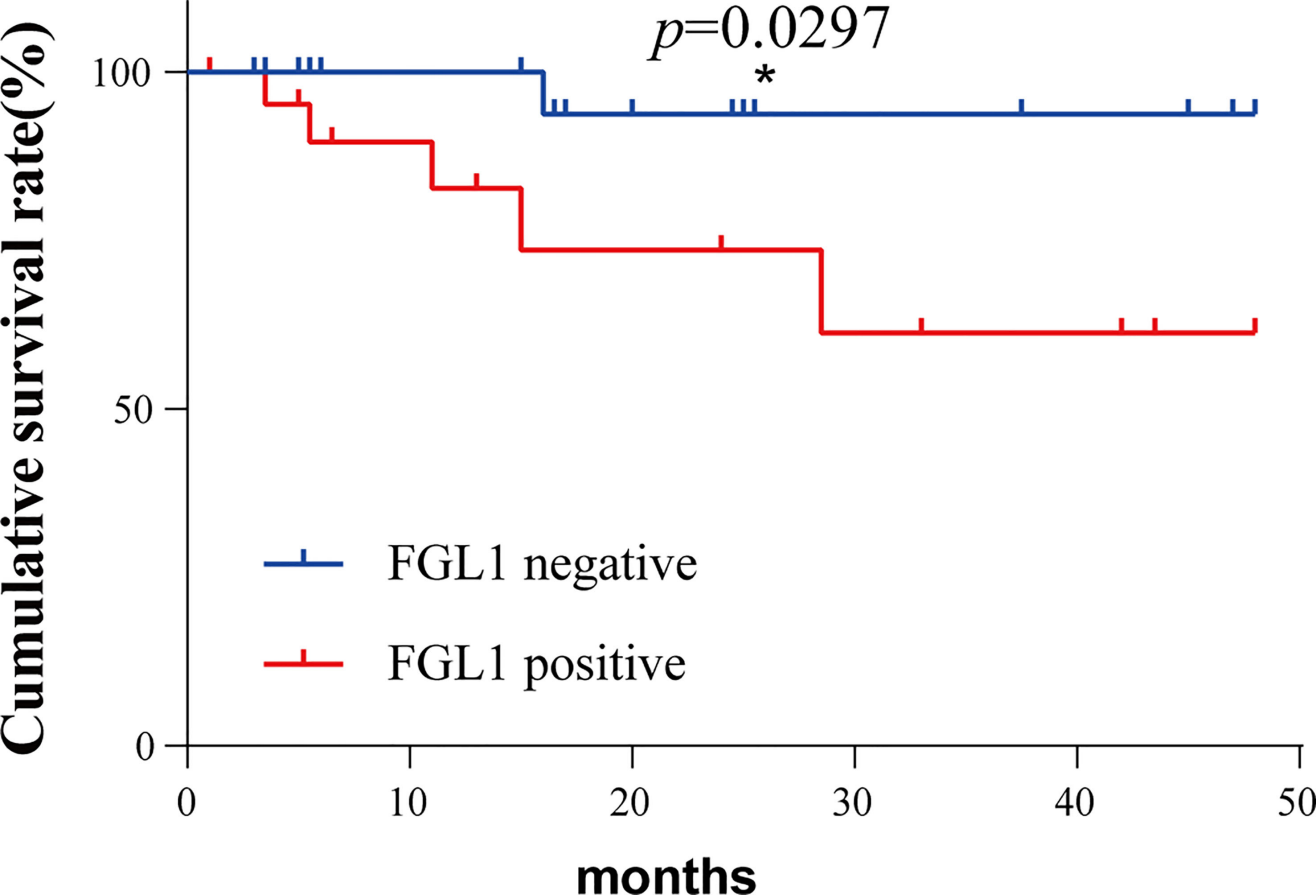
The FGL1 positive of circulating tumor cells was positively correlated with the poor prognosis of patients. *p < 0.05.

The postoperative survival of the matched two groups of patients was analyzed by survival analysis, and the results were shown in **Figure 3**. Patients in the FGL1 positive group have worse postoperative survival than the negative group (p=0.0297). Patients in the FGL1 positive group had a 1-year survival rate of 82.7% and a 3-year survival rate of 61.3%; while those in negative group had a rate of 100% and 93.7%, respectively; The 1-year and 3-year postoperative survival rates of positive group were lower than those in the FGL1 negative group.

### FGL1 Expression Is Higher in Mixed CTCs, and the Number of Mixed CTCs Is Related to the Prognosis of Patients

**Figure 4:**
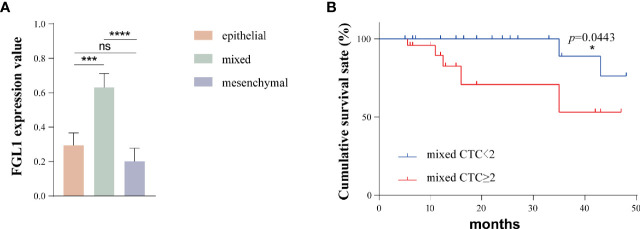
The FGL1 expression level of mixed CTC is higher and the number of mixed CTC is positively correlated with the poor prognosis of patients. **(A)** FGL1 expression levels of different types of CTC; **(B)** Survival analysis of the number of mixed CTCs and patient prognosis. *p < 0.05; ***p < 0.001; ****p < 0.0001; ns, no statistical significance.

A total of 93 patients had epithelial-mesenchymal transition (85%). In order to further explore the FGL1 expression level of different types of circulating tumor cells, we assign a value to the FGL1 expression level of each CTC. No, low, medium and high FGL1 expression were 0,1,2,3 points, respectively. The calculated formula of the average FGL1 expression was the sum of cell number multiplied by expression level/total number of CTCs, which was applied to all types of CTCs in all patients in the previous table. We next used Prism software to map and analyze the results as shown in **Figure 4A**. The FGL1 expression level of mixed CTCs was higher than that of epithelial CTCs (P=0.0002) and mesenchymal CTCs (p<0.0001), while there were not statistically different (p=0.7521).

We further analyzed the relationship between the number of mixed CTCs in the peripheral blood and the prognosis of the patients. With the threshold of 2/5 ml for mixed CTC, the patients were divided into 2 groups, and the propensity score matching method was used. A total of 34 pairs of patients were obtained after 1:1 matching and there was no statistical difference in the clinical indicators between the two groups. The postoperative survival of these two groups was shown in **Figure 4B**. The prognosis of patients in the mixed CTC≥2/5ml group was worse than that of the patients with mixed CTC<2/5ml (p=0.0443).

### Correlation Analysis of FGL1 Expression in CTC and Tissues

**Figure 5:**
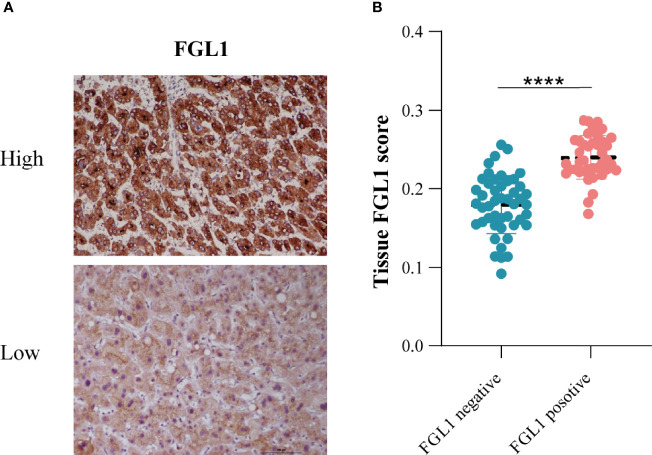
The relationship between tissue FGL1 and CTC FGL1 expression **(A)** The representative figure of tumor tissue FGL1 immunohistochemistry. **(B)** Correlation analysis between tumor tissue and CTC FGL1 expression. ****p < 0.0001.

In addition, we performed FGL1 staining of tumor tissue sections and assessed the FGL1 expression with ImageJ software. The analysis results indicated that the level of FGL1 expression in the tissues of patients with FGL1-positive CTC was significantly higher than that of negative group (**Figure 5**).

### The Relationship Between FGL1 Positive and the Efficacy of Immunotherapy

Among these patients, 12 patients received PD-1/PD-L1 immunotherapy. FGL1 was detected in the circulating tumor cells of 10(83.3%) of them, and 8 of these 10 patients did not respond to immunotherapy.

## Discussion

HCC is currently one of the most threatening diseases to human life and health in the world. The incidence of HCC has been rising in recent years, and the mortality rate has also remained high (1, 2). Despite the development of medicine science, the treatment of advanced HCC is still extremely limited, leaving slight chance for those patients. The overall five-year survival rate of HCC patients is merely 12%-18% (24). In recent years, with the in-depth research on the pathogenesis of HCC, there have been certain breakthroughs in the treatment of advanced HCC. A great progress has also been made in clinical trials related to immune checkpoints. However, current immunotherapy for HCC still faces intractable problems including a low response rate and drug resistance. In order to improve the efficacy of immunotherapy, it is extremely important to find new immune checkpoints and correctly screen patients suitable for immunotherapy. FGL1 is newly discovered as the major ligand of LAG-3. It can bind to LAG-3 to inhibit anti-tumor immunity. This article aimed to detect the expression of FGL1 through circulating tumors, study the significance of FGL1 expression in CTC of HCC patients and explore whether it can be used to predict the efficacy of PD-1/PD-L1 immunotherapy.

To predict the influence of FGL1 on the immune cell infiltration level in various tumors, we performed the bioinformatic analysis to analyze the expression level changes of FGL1 in several tumors in the TCGA database and found that its expression was different between tumor tissue and normal tissue among tumors. The result showed that the expression of FGL1 in various malignant tumors were different in tumor tissues from normal tissues. Furthermore, we performed the correlation analysis between FGL1 expression and immune microenvironments. The result showed that FGL1 expression was correlated with the infiltration of several immune cells in various types of malignancy, which indicates that there might be a relationship between FGL1 expression and tumor immune microenvironments. Further experimental evidences were needed to prove the function of FGL1 in tumors.

Canpatrol ^®^ technique was applied in this study. As it was a new CTC detection method that enriches the CTCs through an 8 um nanomembrane, small size tumor cells might be lost through this method. Also, the level of mRNA doesn’t equals the level of protein expression, this differences needs to be considered. However, this new method also has some advantages. Firstly, the sensitivity of mRNA detection is higher and the background suppression is better. Secondly, the CTC isolation steps are simpler, and the fewer centrifugation and washing steps could help to enhance CTC enrichment.

FGL1 expression was detected in the CTC of 40 (36.70%) of the 109 patients. We further divided the patients into FGL1 positive and negative groups based on the presence or absence of FGL1 expression in the CTC. The results of clinical correlation analysis showed that FGL1 positive patients presented a higher proportion of the advanced TNM stage as well as distant metastases. It suggested that FGL1 expression was related to late-stage carcinoma and distant metastasis, which contributed to the poor prognosis of patients. The survival analysis obtained after propensity score matching demonstrated that the prognosis of FGL1 positive patients was significantly worse compared to the negative. It illustrated that the high expression of FGL1 in CTC was positively correlated with the poor prognosis of HCC patients.

We further studied the expression of FGL1 on different types of CTC, and the results showed that the expression level of FGL1 of mixed CTC was significantly higher than that of epithelial CTC and mesenchymal CTC. Furthermore, patients with a high number of mixed CTCs tended to present a worse prognosis. Previous studies have shown that epithelial-mesenchymal transition (EMT) occurred when tumor cells metastasized to a distant place. The migration and invasion capabilities of tumor cells are enhanced after EMT and tumor cells are more likely to enter the blood circulation, penetrate the blood vessel wall, invade distant tissues and remotely metastasize eventually (25, 26). It has been reported in the literature that only CTC cells with high cell viability and metastatic activity have the ability to eventually develop metastases (27). Mixed CTC has both epithelial and mesenchymal phenotypes, and its ability to survive in the blood is stronger. It has been reported in the literature that mixed CTC may be the type of CTC with the strongest correlation with distant tumor metastasis (28, 29). In this study, patients with a high number of mixed CTCs have a worse prognosis, which is consistent with the EMT theory of distant metastasis of tumors. In addition, the FGL1 expression level of mixed CTC is the highest, and FGL1 positive in circulating tumor cells is associated with distant metastasis and poor prognosis in patients, suggesting that FGL1 expression in HCC tissues may promote EMT of tumor cells, and promote the distant metastasis of HCC. Zhang et al. reported that the expression level of FGL1 in gastric cancer tissues is higher than that in adjacent tissues, and the prognosis of patients with high expression of FGL1 in tumor tissues was worse. Knockdown of FGL1 could significantly increase the E-cadherin level of tumor cells and reduce the expression levels of vimentin and N-cadherin in tumor tissues, suggesting that FGL1 could promote the EMT process of gastric cancer (30).

Based on the above results, we believe that the high expression of FGL1 in HCC tissues and CTC is related to the poor prognosis of patients. Patients with high FGL1 expression are more likely to have distant metastases, which may be related to the mixed CTC with high FGL1 expression in the blood, and therefore the prognosis is worse.

As a newly discovered potential immunotherapy target, the strategy of FGL1 application remains to be further discussed, whether it is used alone or in combination with PD-1/PD-L1 inhibitors to enhance the efficacy. Previous studies have confirmed that FGL1 is the main ligand of LAG-3 and its function of inhibiting the anti-tumor T cells by binding to it. Blocking FGL1 can enhance the anti-tumor immune function of T cells and further inhibit tumor growth, and such anti-tumor effects could even be strengthened in combination with PD-L1 inhibitors (14, 15). In this study, 10 out of 12 patients who received PD-1/PD-L1 immunotherapy showed, FGL1 expression in CTCs, 8 of which did not respond to PD-1/PD-L1 immunotherapy. It suggested that FGL1-positive CTC may be related to immunotherapy tolerance. The detection of circulating tumor cells in the peripheral blood may serve as a promising biomarker for clinical decision of immunotherapy in the near future. We believe that even in these patients with no response to PD-1/PD-L1 inhibitors, FGL1/LAG-3 is involved in the suppression of anti-tumor immunity and results in therapeutic effect. However, the sample size of immunotherapy patients in this article was too small, clinical studies with larger samples are still needed to further confirm the relationship between FGL1 and immunotherapy.

In addition, we performed FGL1 staining on postoperative tissue sections of patients to analyze the relationship between tissue FGL1 expression levels and circulating tumor cell FGL1 expression levels. The results showed that the level of tissue FGL1 in patients with CTC FGL1 positive was significantly higher than that of CTC FGL1 negative patients. This suggests that the FGL1 level of CTC can reflect the expression of FGL1 in the primary tumor. However, because that most patients have no expression of FGL1 on CTCs, we couldn’t use the scattered plot to analyze the relationship between tissue and CTCs FGL1 expression. Strati et al. reported that the PD-L1 expression level of circulating tumor cells in the peripheral blood of patients with head and neck squamous cell carcinoma is related to the patient’s response to PD-L1 inhibitor therapy (31). Janning et al. reported that in non-small cell lung cancer, the detection of the PD-L1 level of circulating tumor cells can predict the patient’s response to PD-L1 inhibitors, and the increased expression of PD-L1 levels of circulating tumor cells in patients after treatment can reflect the patient’s response The resistance of PD-L1 (20). In the future, it may be possible to screen the beneficiaries of FGL1 inhibitors by detecting the expression of CTC FGL1. Compared with tissue samples, the acquisition and detection of CTC has the characteristics of easier, reproducible and less traumatic. In the future, this technology will play an important role in the guidance of immunotherapy and drug monitoring.

In summary, we found that FGL1 may play an important role in the immune microenvironment regulation of several tumors. This article detected the expression of FGL1 in circulating tumor cells of HCC patients and found that its expression was related to the poor prognosis. FGL1 may be able to promote the distant metastasis of HCC and lead to PD-1/PD-L1 immunotherapy tolerance. In addition, we have also found that the detection of peripheral blood CTC can reflect the FGL1 expression level of the primary tumor. In the future, it may serve as an alternative method of tissue sample detection to help clinical decision-making for HCC patients.

## Data Availability Statement

The original contributions presented in the study are included in the article/**Supplementary Material**. Further inquiries can be directed to the corresponding authors.

## Ethics Statement

The studies involving human participants were reviewed and approved by The Ethics Committee of Sun Yat-sen Memorial Hospital. The patients/participants provided their written informed consent to participate in this study.

## Author Contributions

CL, RZ, and L-bX designed the article. QY and H-ML drafted the manuscript and prepared the figures. Z-YZ helped perform the immunohistochemistry. YC and X-LX helped to provide the clinical samples. KZ helped modify the manuscript. All authors read and approved the final manuscript.

## Funding

This work was supported by The Special Research Foundation of the National Nature Science Foundation of China (81972262, 81972255, 81772597, 82173195), The Guangdong Basic and Applied Basic Research Foundation (2020A1515010117); Grant [2013]163 from Key Laboratory of Malignant Tumor Molecular Mechanism and Translational Medicine of Guangzhou Bureau of Science and Information Technology; Grant KLB09001 from the Key Laboratory of Malignant Tumor Gene Regulation and Target Therapy of Guangdong Higher Education Institutes; Grant from Guangdong Science and Technology Department (2015B050501004, 2017B030314026).

## Conflict of Interest

The authors declare that the research was conducted in the absence of any commercial or financial relationships that could be construed as a potential conflict of interest.

## Publisher’s Note

All claims expressed in this article are solely those of the authors and do not necessarily represent those of their affiliated organizations, or those of the publisher, the editors and the reviewers. Any product that may be evaluated in this article, or claim that may be made by its manufacturer, is not guaranteed or endorsed by the publisher.
