# Supplementary material for: Immune Checkpoint FGL1 Expression of Circulating Tumor Cells Is Associated With Poor Survival in Curatively Resected Hepatocellular Carcinoma
Source: Front Oncol. 2022 Feb 22;12:810269. doi: 10.3389/fonc.2022.810269 (PMC8901582; doi:10.3389/fonc.2022.810269)
Supplement: Supplementary file 1 [file Table_1.docx]

| Table S1 CTC fluorescence probe sequence | |
| --- | --- |
| CD45 | TCGCAATTCTTATGCGACTCTGTCATGGAGACAGTCATGTGTATTTCCAGCTTCAACTTCCCATCAATATAGCTGGCATTTTGTGCAGCAATGTATTTCCTACTTGAACCATCAGGCATC |
| EpCAM | TGGTGCTCGTTGATGAGTCAAGCCAGCTTTGAGCAAATGAAAAGCCCATCATTGTTCTGGCTCTCATCGCAGTCAGGATCTCCTTGTCTGTTCTTCTGACCTCAGAGCAGGTTATTTCAG |
| CK8 | CGTACCTTGTCTATGAAGGAACTTGGTCTCCAGCATCTTGCCTAAGGTTGTTGATGTAGCCTGAGGAAGTTGATCTCGTCCAGATGTGTCCGAGATCTGGTGACCTCAGCAATGATGCTG |
| CK18 | AGAAAGGACAGGACTCAGGCGAGTGGTGAAGCTCATGCTGTCAGGTCCTCGATGATCTTGCAATCTGCAGAACGATGCGGAAGTCATCAGCAGCAAGACGCTGCAGTCGTGTGATATTGG |
| CK19 | CTGTAGGAAGTCATGGCGAGAAGTCATCTGCAGCCAGACGCTGTTCCGTCTCAAACTTGGTTCTTCTTCAGGTAGGCCAG  CTCAGCGTACTGATTTCCTCGTGAACCAGGCTTCAGCATC |
| Vimentin | GAGCGAGAGTGGCAGAGGACCTTTGTCGTTGGTTAGCTGGCATATTGCTGACGTACGTCAGAGCGCCCCTAAGTTTTTAAAAGATTGCAGGGTGTTTTCGGGCCAATAGTGTCTTGGTAG |
| Twist | ACAATGACATCTAGGTCTCCCTGGTAGAGGAAGTCGATGTCAACTGTTCAGACTTCTATCCCTCTTGAGAATGCATGCATTTTCAGTGGCTGATTGGCACTTACCATGGGTCCTCAATAA |
| FGL1 | GTTTGGTCTTCCCATTAAACCTCCATTAATCGTATTGTGTGTAAAAACGAAGTCCAGCTGGTCAGAGCGGTGGTAACAAGGCTTCCAAGATCAATGACAGACAGAAAATTCTGCTGGGCTAGTGTAGTCTTCTTGAGTGGGCTGTAGTATACACCATTCA |

| Table S2 TCGA abbreviations and full name | |
| --- | --- |
| Abbr | Full name |
| ACC | Adrenocortical carcinoma |
| BLCA | Bladder Urothelial Carcinoma |
| BRCA | Breast invasive carcinoma |
| CESC | Cervical squamous cell carcinoma and endocervical adenocarcinoma |
| CHOL | Cholangiocarcinoma |
| COAD | Colon adenocarcinoma |
| DLBC | Lymphoid Neoplasm Diffuse Large B-cell Lymphoma |
| GBM | Glioblastoma multiforme |
| HNSC | Head and Neck squamous cell carcinoma |
| KICH | Kidney Chromophobe |
| KIRC | Kidney renal clear cell carcinoma |
| KIRP | Kidney renal papillary cell carcinoma |
| LGG | Brain Lower Grade Glioma |
| LIHC | Liver hepatocellular carcinoma |
| LUAD | Lung adenocarcinoma |
| LUSC | Lung squamous cell carcinoma |
| MESO | Mesothelioma |
| OV | Ovarian serous cystadenocarcinoma |
| PAAD | Pancreatic adenocarcinoma |
| PRAD | Prostate adenocarcinoma |
| READ | Rectum adenocarcinoma |
| SARC | Sarcoma |
| SKCM | Skin Cutaneous Melanoma |
| STAD | Stomach adenocarcinoma |
| TGCT | Testicular Germ Cell Tumors |
| THCA | Thyroid carcinoma |
| THYM | Thymoma |
| UCEC | Uterine Corpus Endometrial Carcinoma |
| UCS | Uterine Carcinosarcoma |
| UVM | Uveal Melanoma |

| Table S3 CTC classification and expression in FGL1 positive patients | | | | | | | | | | | | | |
| --- | --- | --- | --- | --- | --- | --- | --- | --- | --- | --- | --- | --- | --- |
| patients | CTC  total  number | FGL1 positive CTC number | | | | | | | | | | | |
|  |  | Epithelial type | | | | Mixed type | | | | Mesenchymal type | | | |
|  |  | no | low | medium | high | no | low | medium | high | no | low | medium | high |
| 1 | 16 | 0 | 0 | 0 | 0 | 3 | 2 | 0 | 0 | 10 | 1 | 0 | 0 |
| 2 | 1 | 0 | 0 | 0 | 0 | 0 | 1 | 0 | 0 | 0 | 0 | 0 | 0 |
| 3 | 3 | 1 | 0 | 0 | 0 | 0 | 2 | 0 | 0 | 0 | 0 | 0 | 0 |
| 4 | 23 | 2 | 2 | 1 | 0 | 2 | 10 | 6 | 0 | 0 | 0 | 0 | 0 |
| 5 | 27 | 5 | 3 | 0 | 0 | 9 | 8 | 0 | 0 | 1 | 1 | 0 | 0 |
| 6 | 4 | 1 | 0 | 0 | 0 | 2 | 1 | 0 | 0 | 0 | 0 | 0 | 0 |
| 7 | 36 | 2 | 1 | 0 | 0 | 8 | 14 | 9 | 1 | 1 | 0 | 0 | 0 |
| 8 | 4 | 0 | 3 | 0 | 0 | 1 | 0 | 0 | 0 | 0 | 0 | 0 | 0 |
| 9 | 2 | 0 | 0 | 0 | 0 | 0 | 1 | 0 | 0 | 1 | 0 | 0 | 0 |
| 10 | 8 | 0 | 0 | 0 | 0 | 2 | 4 | 2 | 0 | 0 | 0 | 0 | 0 |
| 11 | 9 | 2 | 0 | 0 | 0 | 1 | 3 | 1 | 1 | 1 | 0 | 0 | 0 |
| 12 | 4 | 0 | 0 | 0 | 0 | 2 | 1 | 0 | 0 | 1 | 0 | 0 | 0 |
| 13 | 3 | 0 | 1 | 0 | 0 | 0 | 0 | 0 | 0 | 2 | 0 | 0 | 0 |
| 14 | 1 | 0 | 0 | 0 | 0 | 0 | 0 | 0 | 0 | 0 | 1 | 0 | 0 |
| 15 | 6 | 1 | 0 | 0 | 0 | 2 | 2 | 1 | 0 | 0 | 0 | 0 | 0 |
| 16 | 12 | 3 | 0 | 0 | 0 | 7 | 2 | 0 | 0 | 0 | 0 | 0 | 0 |
| 17 | 15 | 1 | 0 | 0 | 0 | 5 | 9 | 0 | 0 | 0 | 0 | 0 | 0 |
| 18 | 2 | 1 | 0 | 0 | 0 | 0 | 1 | 0 | 0 | 0 | 0 | 0 | 0 |
| 19 | 13 | 2 | 0 | 0 | 0 | 6 | 1 | 0 | 0 | 4 | 0 | 0 | 0 |
| 20 | 18 | 1 | 11 | 0 | 0 | 4 | 2 | 0 | 0 | 0 | 0 | 0 | 0 |
| 21 | 17 | 0 | 0 | 0 | 0 | 4 | 10 | 3 | 0 | 0 | 0 | 0 | 0 |
| 22 | 15 | 0 | 0 | 0 | 0 | 2 | 2 | 0 | 0 | 7 | 3 | 1 | 0 |
| 23 | 11 | 1 | 0 | 0 | 0 | 1 | 0 | 0 | 0 | 8 | 1 | 0 | 0 |
| 24 | 8 | 0 | 0 | 0 | 0 | 3 | 0 | 1 | 0 | 4 | 0 | 0 | 0 |
| 25 | 36 | 0 | 2 | 1 | 2 | 0 | 4 | 13 | 10 | 0 | 0 | 2 | 2 |
| 26 | 9 | 0 | 0 | 0 | 0 | 4 | 1 | 0 | 0 | 4 | 0 | 0 | 0 |
| 27 | 8 | 1 | 1 | 0 | 0 | 0 | 5 | 1 | 0 | 0 | 0 | 0 | 0 |
| 28 | 14 | 4 | 0 | 0 | 0 | 8 | 2 | 0 | 0 | 0 | 0 | 0 | 0 |
| 29 | 10 | 3 | 1 | 0 | 0 | 2 | 2 | 0 | 0 | 1 | 0 | 1 | 0 |
| 30 | 19 | 5 | 2 | 0 | 0 | 7 | 1 | 0 | 0 | 4 | 0 | 0 | 0 |
| 31 | 3 | 0 | 0 | 0 | 0 | 2 | 1 | 0 | 0 | 0 | 0 | 0 | 0 |
| 32 | 3 | 0 | 0 | 0 | 0 | 2 | 1 | 0 | 0 | 0 | 0 | 0 | 0 |
| 33 | 1 | 0 | 0 | 0 | 0 | 0 | 1 | 0 | 0 | 0 | 0 | 0 | 0 |
| 34 | 24 | 7 | 3 | 1 | 0 | 6 | 0 | 6 | 0 | 0 | 1 | 0 | 0 |
| 35 | 23 | 3 | 0 | 0 | 1 | 2 | 1 | 0 | 1 | 13 | 2 | 0 | 0 |
| 36 | 2 | 1 | 0 | 0 | 0 | 0 | 1 | 0 | 0 | 0 | 0 | 0 | 0 |
| 37 | 1 | 0 | 1 | 0 | 0 | 0 | 0 | 0 | 0 | 0 | 0 | 0 | 0 |
| 38 | 19 | 4 | 2 | 0 | 0 | 8 | 1 | 0 | 0 | 3 | 1 | 0 | 0 |
| 39 | 79 | 1 | 4 | 0 | 0 | 3 | 31 | 32 | 5 | 1 | 1 | 1 | 0 |
| 40 | 8 | 0 | 1 | 0 | 0 | 3 | 1 | 0 | 0 | 3 | 0 | 0 | 0 |

| Table S4 The results of univariate COX regression | | | |
| --- | --- | --- | --- |
| characteristics | P value | Hazard Ratio | 95% CI |
| Age | 0.251 | 0.562 | 0.210-1.504 |
| Gender | 0.397 | 0.417 | 0.055-3.158 |
| HbsAg | 0.124 | 4.936 | 0.646-37.730 |
| AFP(ng/ml) | 0.409 | 0.621 | 0.200-1.927 |
| Tumor diameter | 0.358 | 0.628 | 0.234-1.691 |
| Tumor lesion | 0.282 | 1.789 | 0.619-5.169 |
| Lymph node metastasis | 0.597 | 0.579 | 0.076-4.388 |
| Metastasis | 0.314 | 0.039 | 0.001-21.287 |
| TNM stage | 0.266 | 1.745 | 0.654-4.654 |
| FGL1 expression | 0.339 | 0.562 | 0.210-1.504 |

| Table S5 The results of multivariate Cox proportional hazards models | | | |
| --- | --- | --- | --- |
| characteristics | P value | Hazard Ratio | 95% CI |
| Age | 0.185 | 0.483 | 0.164-1.418 |
| Gender | 0.701 | 1.574 | 0.155-15.972 |
| HbsAg | 0.087 | 7.770 | 0.740-81.554 |
| AFP(ng/ml) | 0.096 | 0.242 | 0.046-1.286 |
| Tumor diameter | 0.540 | 0.662 | 0.177-2.475 |
| Tumor lesion | 0.721 | 1.267 | 0.346-4.633 |
| Lymph node metastasis | 0.342 | 0.271 | 0.018-4.020 |
| Metastasis | 0.016 | 4.235 | 0.013-12.537 |
| TNM stage | 0.002 | 8.192 | 2.231-30.077 |
| FGL1 expression | 0.036 | 2.690 | 1.542-5.271 |
